# Association of apolipoprotein E polymorphisms (E2, E3, E4) with type 2 diabetes: a meta-analysis of case-control studies

**DOI:** 10.11604/pamj.2026.53.48.45564

**Published:** 2026-02-03

**Authors:** Chaimae Zouine, Mohamed Amine Ikhanjal, Khaoula Errafii, Hicham Charoute, Meriem Es-Saadi, Fouad Benhnini, Nouha Messoudi, Bouchra Ghazi, Salsabil Hamdi

**Affiliations:** 1 Virology Environmental Health Laboratory, *Institut Pasteur du Maroc*, Casablanca, Morocco,; 2 Immunopathology-Immunotherapy-Immuno monitoring Laboratory, Faculty of Medicine, Mohammed VI University of Health and Sciences, Casablanca, Morocco,; 3 Toxicology, Toxicogenomic, and Ecotoxicology Laboratory, Mohammed VI University of Sciences and Health, Casablanca, Morocco,; 4 African Genomic Center (AGC), University Mohamed VI Polytechnic, Bengurir, Morocco,; 5 Research Unit of Epidemiology, Biostatistics and Bioinformatics, *Institut Pasteur du Maroc*, Casablanca, Morocco,; 6 Laboratory of Cellular Signaling, Faculty of Sciences Meknes, Moulay Ismail University, Meknes, Morocco,; 7 Research Laboratory in Oral Biology and Biotechnology, Faculty of Dental Medicine, Mohammed V University in Rabat, Rabat, Morocco

**Keywords:** Alipoprotein E gene, type 2 diabetes, polymorphisms, allele, genetic association study

## Abstract

Type 2 diabetes (T2D) accounts for over 90% of all diabetes cases and results from the interaction of genetic and environmental factors. However, the association between apolipoprotein E (APOE) gene polymorphisms and T2D risk shows considerable variation across different populations. This meta-analysis aims to clarify the association between APOE genotypes and alleles (E2E2, E2E3, E2E4, E3E4, E4E4) and the risk of T2D. Additionally, it examines the association of demographic, clinical, and biochemical parameters with T2D risk in cases and controls. Relevant articles providing genotypic and allelic frequencies of APOE polymorphisms were sourced from Google Scholar, Web of Science, Science Direct, and PubMed databases. These articles focus on peer-reviewed human case-control studies published in English until February 27, 2024. Data on APOE polymorphisms, biochemical, and clinical parameters were extracted. Statistical tests were performed using Review Manager 4.3.1 with results expressed using ORs and 95% CIs. Publication bias and heterogeneity were assessed using the Q test and Egger regression analysis. Thirty-two studies involving 19644 participants. The statistical analysis showed that BMI, SBP, DBP, TC, and LDL-C could potentially indicate a higher risk of T2D in cases compared to controls. Significant associations with T2D were found for the APOE E4E4 genotype (OR =1.94, 95% CI= [1.16, 3.23], P = 0.01, I^2^=75%), and the E4 allele (OR=1.26, 95% CI= [1.11, 1.43], P = 0.0005, I^2^=55%). No significant associations were observed for the E2E2, E2E3, E2E4, and E3E4 genotypes, or the E2 allele (P > 0.05 for all). A significant association between APOE genotype E4E4 and allele E4 with T2D was confirmed in this meta-analysis.

## Introduction

Type 2 diabetes (T2D), also recognized as non-insulin-dependent diabetes, is a metabolic disease involving persistently high blood sugar levels. This is caused by deficiencies in insulin action, secretion and signalling pathways, which are influenced by various factors, including poor diet and physical inactivity [1]. Type 2 diabetes represents a global health emergency of the 21^st^ century, with its prevalence rapidly increasing due to changes in human behaviors and lifestyle [2]. According to the International Diabetes Federation, there were 536.6 million people worldwide with diabetes in 2021; this number could rise to 783.7 million by 2045, and it was the main cause of death for 6.7 million people in 2021 [3].

Understanding genetic factors contributing to T2D susceptibility is crucial for developing effective preventive strategies and reducing its increasing incidence [4]. This comprehension may also improve treatment precision and efficiency [5]. The apolipoprotein E gene (APOE) is among the most extensively studied genes in the human genome [6], and is recognized as a risk factor for T2D development [7]. This gene encodes a protein of 299 amino acids located on chromosome 19q13.2 with three common alleles (E2, E3, E4) [8]. The most common genotypes are the E3E3, known as the “wild type”, with a frequency of 67% between patients and healthy subjects [9]. It is mainly synthesized in the liver and linked with triglyceride-rich lipoproteins to remove their remnants after enzymatic lipolysis in the circulation [10]. Apolipoprotein E plays a major role in cholesterol homeostasis [11]. Different APOE alleles interact differently with lipoprotein receptors, resulting in varied concentrations of cholesterol and triglycerides [12]. This functional variation associates with phenotypic differences in multiple medical traits, including cholesterol levels, cardiovascular health, and risk of Alzheimer's disease [13]. In addition, the synthesis of this protein by macrophages initiates the formation of high-density lipoproteins, which facilitate the reverse transport of cholesterol to the liver, and nervous system, and distribute lipids among cells [14]. This gene is expressed throughout the body-liver, brain, spleen, kidneys, gonads, and adrenal glands, testifying to its importance in various metabolic pathways such as lipoprotein, fat-soluble vitamins, and glucose metabolism, signal transduction, metastasis or angiogenesis [14,15].

New studies have been published in this field since the last published syntheses, providing additional data on various populations and using improved genetic analysis methodologies. This updated meta-analysis is distinguished by its rigorous inclusion criteria, focusing on study quality and methodological standardization. We employed advanced statistical methods, including comprehensive leave-one-out sensitivity analyses and heterogeneity assessments, to evaluate the robustness of associations more thoroughly than in previous syntheses. As well as providing better geographic and ethnic representation, the inclusion of recent studies improves the generalisability of the results. This approach provides more robust estimates of the association between APOE polymorphisms and T2D risk.

This study provides a comprehensive estimation of the association between APOE genetic polymorphisms and T2D by pooling and analyzing relevant studies to assess the significance and reproducibility of this link. The objective of this meta-analysis is to investigate the association between APOE gene variants and T2D in order to determine the significance of this relationship.

## Methods

**Figure 1:**
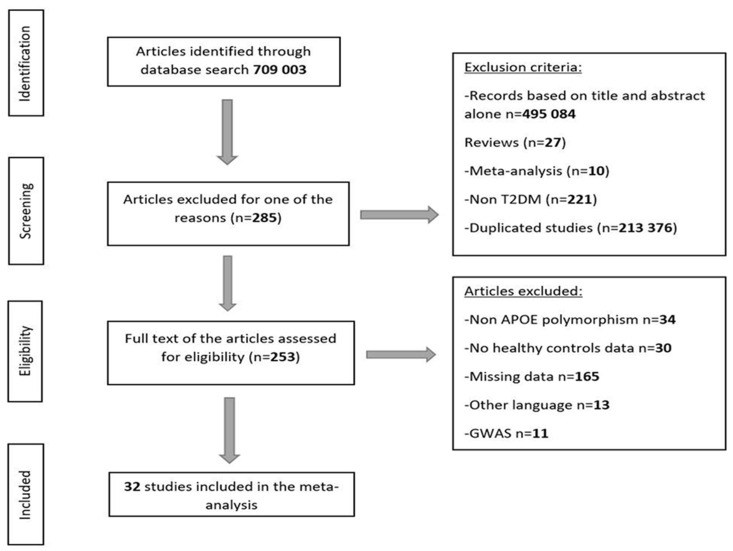
PRISMA flow diagram illustrating the identification, screening, eligibility assessment, and inclusion of studies in this meta-analysis

**Literature search strategy:** PRISMA provided the framework for our search (Figure 1). An exhaustive literature search was conducted across several databases, identifying 30 286 results on ScienceDirect, 676 799 on Google Scholar, 982 on PubMed, and 936 on Web of Science, analysing the association between T2D susceptibility and APOE polymorphism, using the search terms “type 2 diabetes” or “type 2 diabetes mellitus” or “T2D” in combination with the keywords ("APOE polymorphism", “APOE gene” or “APOE SNP”). In this meta-analysis, we included trials from around the world up to 27^th^ February 2024 that examined the association between the APOE gene and T2D susceptibility. Eligible studies were independently identified by reviewers based on pre-defined inclusion and exclusion criteria. In this meta-analysis, only English-language articles were considered eligible.

**Inclusion and exclusion criteria:** the inclusion criteria for this study were defined as follows: 1) Assessment of APOE gene polymorphisms and their association with susceptibility to T2D; 2) case-control study designs; 3) studies in which the sample size and frequency of individual genotypes in cases and controls are available. The studies were excluded if they met one of the following criteria: 1) Meeting abstracts; 2) duplicated articles; 3) meta-analysis; 4) review articles; 5) association between APOE and another type of diabetes, like diabetes type 1 or gestational diabetes; 6) articles missing data on genotypic or allelic frequencies; 7) non-case-control studies; 8) Genome-Wide Association Study (GWAS).

**Data extraction:** the following information was extracted independently from each study included in this meta-analysis: last name of the first author, year of publication, country of the study population, their ethnicities (Asian, Caucasian, Hispanic), number of cases and controls, sample size, mean age, Body Mass Index (BMI), Systolic Blood Pressure (SBP), Diastolic Blood Pressure (DBP), Triglycerides (TG), Total Cholesterol (TC), High-Density Lipoprotein (HDL), Low-Density Lipoprotein (LDL), and the distribution of genotypes and alleles for both cases and controls.

**Statistical analysis:** the data were recorded in a Microsoft Excel spreadsheet and analyzed using Review Manager version 4.3.1. The strength of the association between APOE polymorphisms and T2D was assessed using odds ratios (ORs) with their 95% confidence intervals (CIs), and a p <0.05 was considered statistically significant. A genotype is considered a risk factor if the OR is >1, and as a protective factor if the OR is <1, provided that the 95% CI does not include the value one (OR = 1).

Significance of heterogeneity was determined at a P < 0.10, and by using the I^2^ test. If the I^2^ values were < 50%, the statistical heterogeneity was considered low, and if I^2^ > 50%, it was considered high [16]. Therefore, the random and fixed models were applied accordingly. In this study, we used the Random effects for all APOE polymorphisms (E2E2, E2E3, E2E4, E3E4, E4E4, E2, and E4) when we compared with E3E3 and E3.

We also performed a subgroup analysis stratified according to the ethnic origin of the subjects using the χ^2^-based Q test, and statistical significance for heterogeneity was set at P ≤0.1. When heterogeneity was observed, the pooled OR was estimated for each study using the random-effects model (REM). Otherwise, the fixed-effects model (FEM) was utilized.

The publication bias in the meta-analysis was assessed by Egger´s test [17]. We evaluated the symmetry of the forest plot using Egger's linear regression test to determine the absence of publication bias. A significance level of P < 0.05 was considered statistically significant.

A leave-one-out sensitivity analysis was conducted in Review Manager 4.3.1, whereby each study was excluded in turn and the pooled OR, 95% CI and heterogeneity (I^2^) were re-evaluated for each comparison. Studies whose exclusion substantially altered the results (a change in significance or an OR variation of more than 10%) were considered influential. The results of the included studies were graphically represented by forest plots, which were also generated using Review Manager.

## Results

**Eligible studies and their characteristics:** the current research meets the PRISMA statement guidelines. The Search on PubMed, ScienceDirect, Google Scholar, and Web of Science revealed a total of 709004 articles; we screened the identified articles to select those containing pertinent data. Four hundred ninety-five thousand and eighty-four studies were excluded from the analysis due to lack of relevance, as they only mentioned search terms such as diabetes and APOE in the title or abstract, without being related to our inclusion criteria, and 213376 studies for duplication. After careful screening, 512 articles were excluded for the following reasons: reviews, meta-analyses, not on APOE polymorphism and T2D, not for APOE polymorphism, no healthy control, other languages, and missing data. Finally, thirty-two eligible studies involving 19644 individuals (8743 cases (3678 females and 3709 males) and 10901 controls (4832 females and 4068 males)) were included in our meta-analysis (Figure 1).

**Figure 2:**
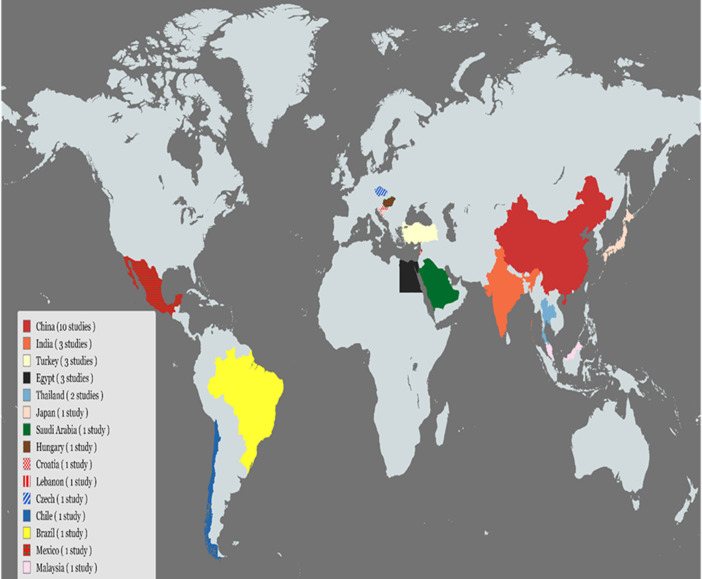
geographic distribution of studies assessing the association between APOE polymorphisms and T2D

These studies have examined the influence of the APOE gene polymorphism on the susceptibility to T2D. We summarized the findings of the allelic and genotypic distributions of each case-control study, country, ethnicity, and sample size in Table 1, Table 2, and Table 3. A total of 32 studies conducted in different countries are included in this analysis [17-48]. Ten studies were performed in the population of China, three from India, two studies were carried out in Thailand, three studies were conducted in Egypt, three in Turkey and one study was performed in each of the following countries: Japan, Hungary, Chile, Brazil, Croatia, Saudi Arabia, Lebanon, Mexico, Czech Republic, Malaysia, and Iraq (Figure 2).

**Table 1:** demographic characteristics of patients with T2D and healthy control individuals, from 32 studies included in this meta-analysis published up to 27^th^ February 2024 (N = 19,644)

Reference	Country	Ethnicity	Cases/ controls	Average age cases/ controls	Gender cases F/ M	Gender controls F/ M
Eto, 1986	Japan	Asian	105/ 111	52±2/ 48±2	-	-
Inamdar, 2000	India	Caucasian	60/ 40		25/ 35	15/25
Kalina, 2002	Hungary	Caucasian	298/ 199	77.7±7.5/-	179/ 119	123/76
Liu, 2003	China	Asian	298/ 87	63.7 ± 12.1/ 66.5 ± 5.3	148/ 150	52/35
Leiva, 2005	Chile	Hispanic	193/ 139	59.5 ± 10.4/ 37.8 ±10.5	93/ 100	104/35
Dixit, 2005	India	Caucasian	136/ 264	46.96 ±9.38/ 47.39 ± 16.64	57/ 79	148/ 116
Errera, 2006	Brazil	Hispanic	95/ 107	-	-	-
Singh, 2006	India	Caucasian	90/ 97	51.68 ±12.58/ 52.10 ±12.67	58/ 32	-
Erdogan, 2009	Turkey	Caucasian	56/ 35	52.60±8.14/ 59.39±11.12	22/ 34	16/ 19
Guan, 2009	China	Asian	213/ 111	70.3 ± 11.3/ 67.7 ± 16.8	144/ 99	55/56
Tascilar, 2009	Turkey	Caucasian	85/ 77	61.67± 13.57/ 54.70± 8.44	34/ 51	52/25
Mustapic, 2012	Croatia	Caucasian	196/ 456	64.8± 9.3/ 77.4± 5.4	56/ 140	186/270
Chaudhary, 2012	Thailand	Asian	155/ 149	51.95 ±0.53/ 52.01 ±0.62	98/ 57	100/49
Atta, 2016	Egypt	Caucasian	45/ 45	56.5±6/ 55.36±7	24/ 21	18/27
Mehmet, 2016	Turkey	Caucasian	50/ 50	58.92± 8,48/ 57.9±54.68	24/ 26	25/25
El Lebedy, 2016	Egypt	Caucasian	100/ 84	50.9 ± 7.5/ 51.8 ± 5.2	43/ 57	39/45
Srirojnopkun, 2018	Thailand	Asian	241/ 275	59.35 ± 9.05/ 47.29± 7.97	183/ 58	196/79
Liu, 2019	China	Asian	247/ 211	61.52 ± 11.61/ 60.65 ± 11.72	119/ 128	102/109
Tang, 2019	China	Asian	156/ 145	62.89 ± 8.38/ 64.10 ± 9.49	71/ 85	67/78
Atageldiyeva, 2019	Lebanon	Caucasian	1422/ 1389	60.1 ± 11.0/ 57.5 ± 10.8	766/ 656	782/607
Rahman, 2019	Malaysia	Asian	51/ 51	71.1±11.3/ 69.2 ±9.6	32/ 19	26/25
Gonzalez-Aldaco, 2020	Mexico	Hispanic	168/ 449	-	105/ 63	303/146
Galal, 2021	Egypt	Caucasian	100/ 100	31.8 ± 10.7/ 31.9 ± 10.8	59/ 41	61/39
Wang, 2021	China	Asian	210/ 243	65:41 ± 8.20/ 64:58 ± 10.:21	110/ 100	116/ 127
Khalaf Alharbi, 2021	Saudi Arabia	Caucasian	438/ 460	53.5 ± 10.78/ 46.13± 7.77	-	-
Dlouha, 2021	Czech	Caucasian	1274/ 2055	61.9 ± 10.3/ 47.9 ± 10.6	491/ 783	1130/ 925
Liu, 2022	China	Asian	150/ 150	56.0 ±47.5/ 63.5±54.0	58/ 92	90/ 60
Wu, 2022	China	Asian	264/545	69.5 ± 12.7/ 67.2 ± 13.4	85/ 179	206/ 339
Zeng, 2023	China	Asian	743/ 995	-	393/ 350	543/ 452
Gan, 2023	China	Asian	306/ 483	60.85 ± 11.35/ 58.83 ± 12.09	181/ 125	247/ 236
Han, 2023	China	Asian	808/ 1226	54.2 ± 2.66/ 80.5± 4.21	-	-
Hameed, 2023	Iraq	Caucasian	50/ 73	46.48±9.3/ 43.51±11.47	20/ 30	30/ 43

F: female; M: male

**Table 2:** clinical and biochemical characteristics, including BMI, SBP, DBP, TG, TC, and LDL-C, of participants included in this meta-analysis (N = 19,644)

Reference	BMI cases/ controls	SBP cases/ controls	DBP cases/ controls	TG cases/ controls	TC cases/ controls	HDL cases/ controls
Eto, 1986	-	-	-	-	-	-
Inamdar, 2000	25/ 23	-	-	2.05/ 1.24	6.42/ 5.43	1.14/ 1.14
Kalina, 2002	28.9± 1.5/ 28.0± 1.1	-	-	2.1± 1.9/ 1.2± 0.6	6.3± 1.4/ 5.9± 0.9	1.1± 0.4/ 1.1± 0.5
Liu, 2003	24.1 ± 3.8/ 22.6 ± 3.3	141.6 ± 20.5/ 138.2 ± 19.2	83.4 ± 11.6/ 86.0 ± 11.1	-	-	-
Leiva, 2005	29.2 ± 4.8/ 22.4 ± 2.1	145.2 ± 20.4/ 122.1 ± 5.6	82.2 ± 13.9/ 76.5 ± 4.7	2.4 ± 1.7/ 1.4 ± 0.1	5.5± 1.3/ 4.2 ± 0.6	1.1 ± 0.3/ 1.2 ± 0.3
Dixit, 2005	24.37 ± 4.21/ 23.34 ± 2.68	-	-	-	-	-
Errera, 2006	-	-	-	-	-	-
Singh, 2006	22.85± 4.87/ -	-	-	-	-	-
Erdogan, 2009	29.16±9.34/ 27.12±3.12	120.36±10.78/ 114.36±11.42	75.54±7.11/ 74.36±5.02	4.33±2.19/ 3.24±2.53	5.39±0.95/ 4.47±1.07	2.41±0.27/ 1.34±0.31
Guan, 2009	-	-	-	-	-	-
Tascilar, 2009	29.19± 5.06/ 28.21± 6.48	142.40±30.63/ 127.63±21.32	83.25± 15.04/ 81.80± 10.39	1.84±0.96/ 1.74±1.012	4.95±1.15/ 5.25±0.99	1.06±0.32/ 1.27±0.36
Mustapic, 2012	-	-	-	-	-	-
Chaudhary, 2012	26.89± 0.31/ 23.84 ± 0.27	147.31±1.76/ 111.54±1.12	84.50 ± 1.01/ 73.11± 0.86	2.49 ±0.12/ 1.11 ±0.04	6.15±0.14/ 4.96±0.04	1.3±0.03/ 1.55 ±0.03
Atta, 2016	25 ± 2/ 23 ± 1.3	133 ± 13/ 124 ± 10	83 ± 8.9/ 72 ± 19	1.74 ± 0.8/ 1.38± 0.16	4.84± 1.14/ 3.67± 0.31	-
Mehmet, 2016	29.82 ± 4.48/ 27.86 ± 4.38	128.19 ± 12.94/ 110.40± 11.16	77,10 ±8,62/ 70,10± 7,54	4.37±2.23/ 3.36± 0.16	4.59± 0.94/ 4.53± 0.21	1.05± 0.31/ 1.25± 0.06
El-Lebedy,2016	27.59 ± 4.90/ 23.21 ± 4.62	131.5 ± 19.7/ 115.3 ± 9.6	79.7 ± 18.0/ 77.6 ± 6.9	1.64 ± 0.81/ 1.33 ± 0.35	4.97 ± 1.25/ 4.55± 0.41	1.27 ± 0.29/ 1.4 ± 0.25
Srirojnopkun, 2018	27.15± 13.52/ 23.29± 2.94	139.43±20.59/ 128.27±16.63	78.19 ± 12.77/ 79.49 ± 11.08	1.91 ± 0.75/ 1.2 ± 0.72	6.5 ± 1.06/ 5.65 ± 1.35	1.23 ± 0.32/ 1.38 ±0.33
Liu, 2019	-	141.26± 24.43/ 124.49± 14.90	81.60 ± 15.34/ 77.03 ± 10.75	2.75 ± 3.64/ 1.59 ± 0.93	5.67 ± 1.84/ 4.21 ± 1.29	1.27 ± 0.37/ 1.33 ± 0.43
Tang, 2019	27.32 ± 2.85/ 27.87 ± 3.12	-	-	-	-	-
Atageldiyeva, 2019	27.5 ± 4.2/ 23.4 ± 3.4	141.43 ± 28.6/ 121.5 ±14.1	81 ± 13.6/ 78 ± 10.4	1.7 ± 1.3/ 1.5 ± 0.8	5.1 ± 1.3/ 4.9 ± 1.5	1.1 ± 0.3/ 1.5 ± 0.9
Rahman, 2019	-	131.81±10.13/ 134.58±18.76	79.52±10.52/ 82.55±16.59	-	-	-
Gonzalez-Aldaco, 2020	28.8 ± 5.51/ 22.4 ± 2.2	-	-	2.35 ± 1.31/ 1.30 ± 0.75	5.21 ± 1.32/ 4.72 ± 0.87	1.1 ± 0.28/ 1.27 ± 0.37
Galal, 2021	25.2 ± 1.4/ 24.3 ± 1.8	-	-	1.47 ± 0.46/ 1.03 ± 0.33	5.05 ± 0.78/ 4.70 ± 0.55	1.11 ± 0.13/ 1.1 ± 0.13
Wang, 2021	25.29 ± 3.98/ 25.75 ± 3.19	142.67± 19.41/ 133.98± 18.63	81.64 ± 10.17/ 78.17 ± 10.67	1.88 ± 1.62/ 2.00 ± 1.46	4.20 ± 1.24/ 4.24 ± 1.14	1.05 ± 0.33/ 1.04 ± 0.31
						
Khalaf Alharbi, 2021	29.9 ± 5.89/ 29.22 ± 5.58	125.83 ± 9.96/ 114.80 ± 8.04	81.25 ± 4.82/ 75.81 ± 6.20	2.24 ± 1.62/ 1.62 ± 0.86	5.61± 1.26/ 5.04 ± 0.96	0.84 ± 0.37/ 0.64 ± 0.23
Dlouha, 2021	30.9 ± 5.0/ 27.4± 4.5	-	-	2.07 ± 1.57/ 1.60± 0.99	4.57 ± 1.01/ 5.73± 1.12	-
Liu, 2022	26.60±24.50/ 25.88 ±24.08	-	-	1.56 ±1.16/ 1.63±1.12	5.45±4.88/ 5.12±4.50	1.21±1.02/ 1.22±1.04
Wu, 2022	-	-	-	1.72 ± 1.30/ 1.42 ± 0.91	4.30 ± 1.23/ 4.25 ± 0.96	1.05 ± 0.28/ 1.15 ± 0.33
Zeng, 2023	-	-	-	1.18 ± 0.86/ 1.21 ± 0.87	4.54 ± 3.65/ 4.25 ± 3.55	1.04 ± 0.85/ 1.07 ± 0.87
Gan, 2023	-	141± 35.3/ 134± 29	79 g±16/ 80 ±17	1.49 ±1.56/ 1.39 ±1.16	4.82 ± 1.37/ 4.85 ± 1.11	1.14±0.42/ 1.19 ±0.41
Han, 2023	-	-	-	2.20±2.03/ 1.63±1.40	4.99±1.42/ 4.77±1.07	1.22±0.37/ 1.29±0.37
Hameed, 2023	31.74±6.37/ 28.11±5.98	-	-	-	-	-

T2D: type 2 diabetes; BMI: body mass index; SBP: systolic blood pressure; DBP: diastolic blood pressure; TG: triglycerides; TC: total cholesterol; HDL: high density lipoprotein; LDL: low density lipoprotein

**Table 3:** distribution of APOE genotypes (E2E2, E2E3, E2E4, E3E3, E3E4 and E4E4) and alleles (E2, E3 and E4) in individuals with T2D and in healthy control group

First author, year of publication	Cases (T2D)									Controls								
	Genotypic frequency of cases						Allelic frequency cases			Genotypic frequency controls						Allelic frequency controls		
	E2E2	E2E3	E3E4	E3E3	E3E4	E4E4	E2	E3	E4	E2E2	E2E3	E2E4	E3E3	E3E4	E4E4	E2	E3	E4
Eto, 1986	0	9	0	73	21	2	6	88	11	1	10	1	80	16	3	5	93	13
Inamdar, 2000	2	8	3	17	16	-	-	-	-	1	9	2	10	8	10	-	-	-
Kalina, 2002	-	26	-	233	38	-	26	530	40	-	21		140	37	-	21	338	39
Liu, 2003	1	47	3	193	53	1	49	486	55	0	4	2	64	11	0	4	143	11
Leiva, 2005	0	12	4	133	43	1	16	321	48	0	10	3	87	39	0	13	223	42
Dixit, 2005	1	11	4	101	18	1	17	231	24	0	19	6	197	41	1	25	454	49
Errera, 2006	-	13	2	68	12	-	8	80	7	7	-	0	77	23	0	3	92	12
Singh, 2006	1	4	2	78	5	0	8	165	7	1	7	0	74	13	2	9	168	17
Erdogan, 2009	-	4	0	40	12	-	4	96	12	-	0	0	28	7	-	0	63	7
Guan, 2009	8	32	7	141	24	1	55	338	33	1	11	1	88	9	1	14	196	12
Tascilar, 2009	3	18	3	45	9	7	27	117	26	3	16	7	40	9	2	29	105	20
Mustapic, 2012	0	35	2	127	30	2	37	319	36	1	48	2	328	76	1	52	780	80
Chaudhary, 2012	1	117	4	2	1	30	3	132	20	2	12	0	113	21	1	8	130	11
Atta, 2016	-	12	12	12	9	-	24	45	21		3	3	30	9		6	72	12
Mehmet, 2016	-	2	-	43	5	-	1	45	3	-	22	-	19	9	-	11	34	4
El-Lebedy, 2016	-	-	-	80	12	-	-	-	-				66	7				
Srirojnopkun, 2018	10	9	3	147	65	7	32	368	82	5	41	6	163	56	4	57	423	70
Liu, 2019	2	16	3	183	43	-	20	425	46	1	24		160	25	1	26	369	26
Tang, 2019	1	26	4	79	38	8	32	222	58	3	28	1	95	17	1	35	235	20
Atageldiyeva, 2019	0	198	205	509	434	76	403	1141	715	0	366	184	574	154	111	550	1094	449
Rahman, 2019	0	6	7	22	14	2	13	64	25	0	2	7	27	14	1	9	70	23
Gonzalez-Aldaco, 2020	1	27	2	118	20	0	31	283	22	0	24	5	340	77	3	30	780	88
Wang, 2020	2	39	4	126	37	2	47	328	45	45	28	7	172	31	1	43	403	40
Galal, 2021	18	6	5	46	12	13	47	110	43	12	9	7	58	10	4	40	135	25
Dlouha, 2021	9	143	19	871	234	13	-	-	-	14	258	30	1405	323	25	-	-	-
Khalaf Alharbi, 2021	35	26	13	290	35	39	109	641	126	27	18	11	334	60	10	83	746	91
Liu, 2022	12	12	12	109	29	29	12	257	31	23	23	23	104	22	22	25	250	23
Wu, 2022	3	47	2	171	39	2	55	428	45	6	87	6	387	58	1	105	919	66
Zeng, 2023	4	80	13	452	178	16	101	1162	223	5	123	21	647	181	18	154	1598	238
Gan, 2023	4	59	8	193	40	2	75	485	52	3	33	8	362	72	5	47	829	90
Han, 2023	8	97	17	544	138	4	130	1323	163	7	118	12	903	173	13	144	2097	211
Hameed, 2023	-	6	1	38	5	-	7	87	6	-	5	0	67	1	-	5	140	1

T2D: type 2 diabetes; E2, E3, E4: APOE alleles; E2E2, E2E3, E2E4, E3E3, E3E4, E4E4: APOE genotypes; “_”: data not available

The comparison of clinical and biochemical parameters between cases and controls is presented in Table 4. The findings suggest that the cases exhibit higher BMI, SBP, DBP, TC, and LDL levels compared to controls. These differences could potentially indicate a higher risk of T2D in the cases compared to the control group (Table 4).

**Table 4:** comparison of clinical and biochemical characteristics of patients with T2D and healthy control individuals across the studies included in this meta-analysis

Clinical parameters	Controls	Cases	P-value
Age	58.74 ± 9.64	54.40 ± 10.98	0.07
Body mass index	25.24 ± 2.38	27.38 ± 2.38	<0.0001
SBP	123.67± 7.87	136.86 ± 9.04	<0.0001
DBP	77.77 ± 1.78	81.79 ± 3.8	0.001
Triglycerides	1.52 ± 0.68	2.17 ± 0.81	0.16
Total cholesterol	4.61 ± 1.08	4.98 ± 1.26	0.03
HDL	1.20 ± 0.22	1.11 1± 0.11	0.44
LDL	3.03 ± 0.54	3.45 ± 0.70	0.0008

T2D: type 2 diabetes; BMI: body mass index; SBP: systolic blood pressure; DBP: diastolic blood pressure; TG: triglycerides; TC: total cholesterol; HDL: high-density lipoprotein; LDL: low-density lipoprotein

**Quantitative data analysis:** we used Q statistics to test the homogeneity of the effect sizes; a P-value <0.05 indicated acceptance of heterogeneity. In addition, I^2^ was used to measure the size of the heterogeneity. If I^2^ was >50%, it was considered heterogeneous. In our study, all overall effect sizes in genetic models were significantly heterogenous E2E2 (P <0.05 or I^2^=55%), E2E3 (P <0.05, I^2^=87%), E2E4 (P <0.05, I^2^=41%), E3E4 (P <0.05, I^2^=75%), E4E4 (P<.05, I^2^=75%), E2 (P <0.05, I^2^=80%), E4 (P <0.05, I^2^=55%), when comparing with the genotype E3E3 and allele E3. Therefore, the random-effects model was used. The results of the two model analyses are shown in Table 5.

**Table 5:** association between APOE genotypes and alleles and the risk of T2D in Asian, Caucasian, and Hispanic populations, expressed as ORs with 95% confidence intervals

Genetic models	Asian		Caucasian		Hispanic	
	Global OR [CI]	A p-value of Z test	Global OR [CI]	A p-value of Z Test	Global OR [CI]	A p-value of Z test
E2E2	1.17 [0.57, 2.41] R	0.67	1.42 [0.97, 2.08] F	0.07	-	-
E2E3	1.44 [0.92, 2.27] R	0.11	1.03 [0.70, 1.52] R	0.89	1.66 [0.41, 6.66] R	0.48
E2E4	1.09 [0.80, 1.48] F	0.60	1.26 [1.04, 1.53] F	0.02	0.99 [0.32, 3.06]F	0.99
E3E4	1.38 [1.22, 1.55] F	<0.00001	1.14 [0.75, 1.72] R	0.55	0.72 [0.52, 1.01] F	0.06
E4E4	2.03 [0.93, 4.42] R	0.07	2.00 [0.92, 4.36] R	0.08	-	-
E2	1.15 [0.88, 1.50] R	0.30	1.24 [0.84, 1.85] R	0.28	1.88 [0.78, 4.54] R	0.16
E4	1.31 [1.18, 1.45] F	<0.00001	1.32 [1.04, 1.69] R	0.02	0.73 [0.54, 1.00] F	0.05

T2D: type 2 diabetes; OR [CI]: Odds Ratio [Confidence Interval]; P-value (Z test): association test p-value; -value (Q test): heterogeneity test p-value; E2E2, E2E3, E2E4, E3E4, E4E4: genotypes of APOE gene; E2, E4: Alleles of APOE gene

The current meta-analysis compared five genotypes (E2E2, E2E3, E2E4, E3E4, E4E4 and E2, E4) with E3E3 genotype, shows significant association APOE gene polymorphism and T2D under the genotypic model E4E4 (OR = 1.94, 95% CI = 1.16-3.23, P = 0.01), and the allelic model E4 (OR = 1.26, 95% CI = 1.11-1.43, P = 0.0005). However, there was no significant association under: E2E2 (OR = 1.22, 95% CI = 0.77-1.95, P > 0.05), E2E3 (OR = 1.27, 95% CI = 0.95-1.70, P >0.05), E2E4 (OR = 1.26, 95% CI=0.94-1.70, P >0.05), E3E4 (OR = 1.20, 95% CI=0.99-1.44, P >0.05) and the allelic model E2 (OR = 1.23, 95% CI = 1.00-1.53, P = 0.05).

The stratified analyses by ethnicity are shown in Table 5 shown that there was no significant association between APOE and T2D in Asians when we compared all the genetics models with E3E3 except the genotypic model E3E4 (with an OR of 1.38; 95% CI= 1.22-1.55, P <0.05) and allele E4 (with an OR of 1.31; 95% CI= 1.18-1.45, P <0.05). The same for Caucasian ethnicity, there was no association observed under all genetic models in comparison with E3E3 except for the genotypic model E2E4 (with an OR of 1.26; 95% CI= 1.04-1.53, P <0.05) and the allelic model E4 (with an OR of 1.32; 95% CI= 1.04-1.69, P <0.05). For the Hispanic ethnicity, there was no significant association between APOE genetic models and T2D when compared with the allelic model E3.

**Figure 3:**
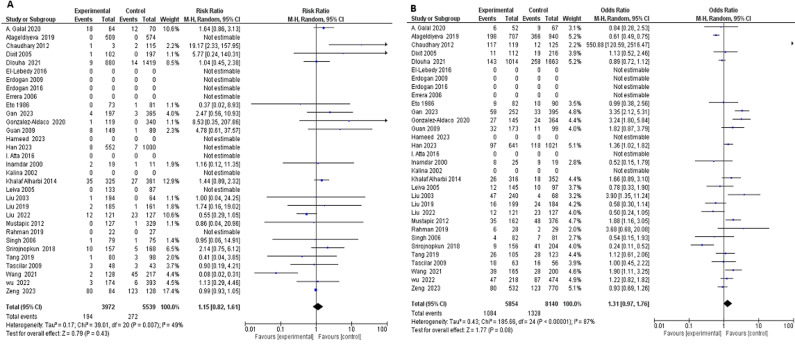
A) forest plot of E3E3 vs E2E2; B) forest plot of E3E3 vs E2E3

**Figure 4:**
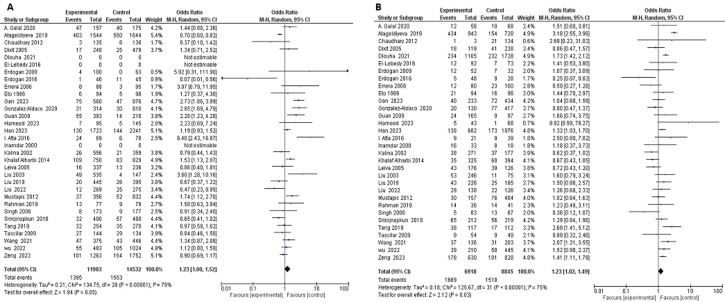
A) forest plot of E3E3 vs E2E4; B) forest plot of E3E3 vs E3E4

**Figure 5:**
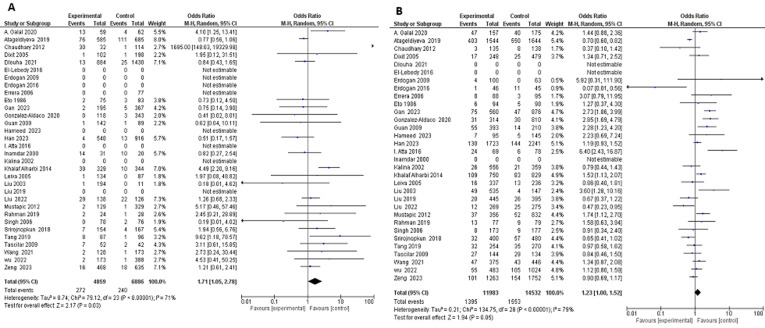
A) forest plot of E3E3 vs E4E4; B) forest plot of E3 vs E2

**Figure 6:**
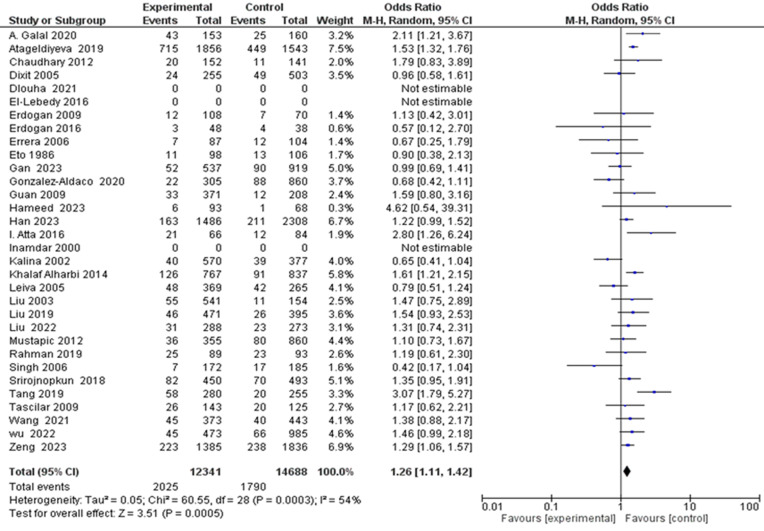
forest plot of E3 vs E4

Subsequently, we conducted an Egger's regression test to evaluate the presence of publication bias across all genetic models, which revealed an absence of statistically significant bias (Table 6). Furthermore, the analysis of the forest plots (Figure 3(A,B), Figure 4(A,B), Figure 5(A,B), Figure 6(A,B)) reveals varying levels of robustness, depending on the comparisons of APOE gene polymorphisms being made. The E3 vs E4 comparison (Figure 6) shows the strongest association (OR = 1.26 [1.11, 1.42], I^2^= 54%, P = 0.0005), with consistency between the included studies and moderate heterogeneity, indicating a 26% increase in the risk of T2D for E4 allele carriers. However, several comparisons have significant limitations. The E3E3 vs E2E3 comparison (Figure 3B) shows excessive heterogeneity (I^2^= 87%) without reaching significance (P= 0.08); the E3 vs E2 comparison (Figure 5B) reveals a weak association bordering on significance (OR = 1.23 (1.00, 1.52)) with high heterogeneity (I^2^= 79%), and the E3E3 vs E2E2 (Figure 3A) comparison shows no significant association (P = 0.43). Although the E3E3 vs E3E4 (Figure 4B) and E3E3 vs E4E4 (Figure 5A) comparisons are statistically significant, they show high heterogeneity (75% and 71%, respectively) and wide CI, which limits the reliability of these associations. These results suggest that the E4 allele is the main identifiable genetic risk factor for T2D in the context of APOE polymorphisms.

**Table 6:** summary of meta-analysis results for the association between APOE genotypes and alleles and the risk of T2D, expressed as ORs, 95% CIs, P-values, and heterogeneity indices I^2^

Genetic models	Test of association		Test of heterogeneity		Effect model	Publication bias
	Global OR [CI]	p-value of Z test	p-value of Q test	I^2^ (%)		P value (Egger's test)
E2E2	1.22 [0.77, 1.95]	0.40	0.003	55	Random	0.8844
E2E3	1.27 [0.95, 1.70]	0.11	<0.00001	87	Random	0.0875
E2E4	1.26 [0.94, 1.70]	0.12	0.01	41	Random	0.9922
E3E4	1.20 [0.99, 1.44]	0.06	<0.00001	75	Random	0.1605
E4E4	1.94 [1.16, 3.23]	0.01	<0.00001	75	Random	0.228
E2	1.23 [1.00, 1.53]	0.05	<0.00001	80	Random	0.1121
E4	1.26 [1.11, 1.43]	0.0005	0.0002	55	Random	0.2948

T2D: type 2 diabetes; OR [CI]: Odds Ratio [Confidence Interval]; I^2^: percentage of heterogeneity; P-value (Z test): association test p-value; -value (Q test): heterogeneity test p-value; random: random-effects model; P value (Egger's test): publication bias test p-value; APOE: Apolipoprotein E

**Sensitivity test:** the leave-one-out sensitivity analysis reveals varying levels of robustness for different APOE polymorphism comparisons. The most robust associations were observed for the E3E3 vs E2E4 comparison (with an OR ranging from 1.17 to 1.35 and no change in significance), and for the E3 vs E4 comparison (with an odds ratio ranging from 1.22 to 1.29 and significance maintained in all exclusions). These results demonstrate excellent stability with moderate heterogeneity (47-55% for the E3 vs E4 comparison). The E3E3 vs E2E2 comparison also yields robust results (OR ranging from 1.12 to 1.37), despite one problematic study. However, three comparisons show worrying fragility: E3E3 vs E2E3 is significant or not depending on the exclusion of studies with persistent heterogeneity (80-88%), E3E3 vs E3E4 loses significance when four critical studies are excluded, and E3 vs E2 is extremely unstable, with 61% of exclusions (17/28) rendering the association non-significant. These results suggest that associations involving the E4 allele (E3E3 vs E2E4 and E3 vs E4) are the most reliable and clinically relevant. In contrast, comparisons involving E2 or E3E4 require cautious interpretation due to their critical dependence on specific studies and high unresolved heterogeneity (Annex).

## Discussion

Diabetes is a serious global public health problem affecting multiple tissues and organs [2,25]. It is a systemic metabolic disorder with the potential to cause long-term dysfunction [49,50]. Mounting evidence suggests that relatives of T2D patients are more likely than the general population to be diagnosed with the disease, and prevalence rates of the condition differ considerably all over the world [35,51]. Identifying novel genetic markers that substantially influence pathogenesis is crucial for the early identification and personalised management of T2D [42,52,53]. The aim of this meta-analysis was to evaluate the association between APOE gene polymorphisms (E2, E3, and E4) and the risk of T2D, synthesising data from 32 case-control studies involving 19644 participants. Our results reveal a robust and significant association for the E3 vs E4 comparison (OR = 1.26 (1.11, 1.42), P <0.001), indicating that carriers of the E4 allele have a 26% higher risk of developing diabetes. Leave-one-out sensitivity analysis confirmed the stability of this association, with OR varying from 1.22 to 1.29. In contrast, other comparisons demonstrate varying degrees of robustness, with some exhibiting significant fragility depending on the specific studies included. Our results revealed a significant correlation between the E4E4 genotype and the E4 allele, indicating an increased diabetes risk.

Apolipoprotein E is a 299 amino acid glycoprotein that binds to the low-density lipoprotein receptor [25]. It is an essential chylomicron apoprotein that interacts with a specific receptor in the liver and peripheral cells [34,43]. However, it is known to control lipoproteins' interaction with other lipoprotein receptors, such as the VLDL receptor and the protein linked to the LDL receptor [28,53]. This finding is consistent with our study, which includes 32 eligible studies involving 19644 individuals, including 8743 cases (3678 females and 3709 males) and 10901 controls (4832 females and 4068 males). The statistical analysis of the clinical and biochemical data shows that the parameters of BMI, SBP, DBP, TC, and LDL were significantly higher in the cases compared to the control group. Existing cross-sectional investigations involving different communities have consistently shown that compared to E3 homozygotes, E2 and E4 carriers had greater triglyceride levels and, respectively, lower and higher levels of blood total and LDL cholesterol [12,54,55]. Additionally, the relationships between APOE alleles and lipid profiles have been validated by a long-term study conducted on a Caucasian population [26,27]. In the linear mixed models, E4 carriers and E3 homozygotes do not exhibit any statistical significance. However, in the logistic mixed models, E4 carriers had a greater probability of having high triglyceride levels (≥1.7 mmol/l) [55,56].

The findings of the present study demonstrate that the presence of the E4 allele and the E4E4 genotype is associated with an increased risk of developing T2D. However, no association was observed between T2D and the E2 allele or the E2E2, E2E3, E2E4, and E3E4 genotypes. Extensive research has been conducted on the role of the APOE gene in the risk of T2D, but the conclusions are still contradictory. However, the majority of studies suggest that the E4 allele is linked to an increased risk of T2D [8]. The present findings are largely consistent with those of a meta-analysis published in 2019, which included 59 studies involving 6872 cases and 8250 controls [56]. This analysis confirmed a significant association between APOE gene polymorphism (E4 allele and E4E4 genotype) and the risk of T2D. Nevertheless, the study revealed no association between the E2E3 and E2E4 genotypes. However, there were minor differences observed for the E2E2 and E3E4 genotypes [43,56]. Moreover, a comprehensive meta-analysis conducted in 2014 [8], encompassing 29 articles including 4615 cases and 2867 controls, revealed a significant association between the E2E3, E3E4, and E4E4 genotypes, as well as the E2 and E4 alleles P < 0.01), and the risk of T2D. But no significant relationship was found for the E2E2, E2E4 genotypes and T2D [9].

However, no relationship has been found between the APOE gene polymorphism and T2D in some studies. For example, a study conducted in 2003 on a Chinese population (74 patients with T2D and 191 healthy controls) showed no significant association between APOE polymorphism and T2D (P> 0.05) [57]. Similarly, a parallel study conducted in northern China involving 150 patients with T2D and 150 patients with T2D associated with lower extremity arterial disease (LEAD) also found no correlation between APOE gene polymorphism and the risk of T2D [34].

The subgroup analysis indicates a significant association in the genotypic model E3E4 (P < 0.05) and allele E4 (P <0.05). The same for Caucasian ethnicity, there was a significant association between the genotypic model E2E4 (P <0.05) and the allelic model E4 (P <0.05). For the Hispanic ethnicity, there was no significant association between APOE genetic models and T2D when compared with the genotypic allelic models E3E3 and E3. These findings suggest that the e4 alleles are associated with an increased risk of T2D. Additionally, this meta-analysis shows no significant heterogeneity in all genetic models. Furthermore, the Egger test was carried out to investigate publication bias in the selected studies. The results indicated that there was no evidence of publication bias.

This study has several notable strengths. These include the rigorous application of leave-one-out sensitivity analyses and the systematic assessment of the robustness of associations across all genetic comparisons. This allowed us to identify fragile versus stable associations. By including all published literature on the association between the APOE gene and T2D from fifteen countries representing diverse ethnicities, we enhanced the generalisability of our findings. Including recent studies increased our statistical significance, with analyses of over 19644 individuals providing more robust evidence of the APOE-T2D association. Additionally, our publication bias analysis revealed no significant bias affecting our results. However, several limitations must be acknowledged. Substantial heterogeneity was observed in certain comparisons (I^2^>70% for E3E3 vs E2E3 and E3 vs E2), suggesting the influence of unmeasured confounding factors, such as environmental exposures, lifestyle factors, and gene-environment interactions, which could not be assessed in this meta-analysis of study-level data. The predominance of studies from certain ethnic populations limits the generalisability of the findings to underrepresented populations. As this was a meta-analysis of published case-control studies, we were unable to adjust for individual-level confounders or examine gene-gene interactions. Variations in diabetes diagnostic criteria and genotyping methods across studies may have contributed to the observed heterogeneity. Despite these limitations, our comprehensive sensitivity analyses and large sample size provide robust evidence of an association between APOE E4 and T2D risk.

## Conclusion

Our meta-analysis found a significant association between the APOE E4E4 genotype, the E4 allele, and an increased risk of T2D. We also identified the following specific associations: E3E4 and E4 in Asian populations and E2E4 and E4 in Caucasian populations. Additionally, correlations were observed between certain demographic, clinical, and biochemical parameters and the risk of T2D. These results suggest that further research is needed in the form of larger, high-quality studies to confirm these associations and explore their mechanisms.

### What is known about this topic

- The APOE gene, located on chromosome 19, encodes a protein of 299 amino acids and comprises 3 alleles (E2, E3, and E4) and six genotypes;
- The E3 allele is the most prevalent and considered neutral, while the E4 allele is considered a risk factor;
- Clinical and biochemical parameters, including mean age, BMI, SBP, DBP, TG, TC, HDL-c, and LDL-c, are associated with T2D.

### What this study adds

- This study examined the relationship between APOE gene polymorphisms and T2D using data from 32 studies involving 16 countries;
- The meta-analysis encompassed 19644 individuals (8743 cases and 10901 controls) and included three major ethnic groups: Asians, Caucasians, and Hispanics;
- The results reveal a significant correlation between the E4 allele and an increased risk of developing T2D.

## Annex

**Annex**: supplementary materials (PDF - 265KB)
